# Effects of DISC1 Polymorphisms on Resting-State Spontaneous Neuronal Activity in the Early-Stage of Schizophrenia

**DOI:** 10.3389/fpsyt.2018.00137

**Published:** 2018-05-23

**Authors:** Ningzhi Gou, Zhening Liu, Lena Palaniyappan, Mingding Li, Yunzhi Pan, Xudong Chen, Haojuan Tao, Guowei Wu, Xuan Ouyang, Zheng Wang, Taotao Dou, Zhimin Xue, Weidan Pu

**Affiliations:** ^1^Mental Health Institute, Second Xiangya Hospital, Central South University, Changsha, China; ^2^Key Laboratory of Psychiatry and Mental Health of Hunan Province, The China National Clinical Research Center for Mental Health Disorders, National Technology Institute of Psychiatry, Changsha, China; ^3^Departments of Psychiatry and Medical Biophysics & Robarts and Lawson Research Institutes, University of Western Ontario, London, ON, Canada; ^4^Zhejiang University School of Medicine, Zhejiang University, Hangzhou, China; ^5^Department of Neurosurgery, The First affiliated Hospital of Xinjiang Medical University, Urumqi, China; ^6^Medical Psychological Center, Second Xiangya Hospital, Central South University, Changsha, China; ^7^Medical Psychological Institute of Central South University, Changsha, China

**Keywords:** schizophrenia, *DISC1*, regional homogeneity, resting-state neuronal activity, genotype

## Abstract

**Background:** Localized abnormalities in the synchrony of spontaneous neuronal activity, measured with regional homogeneity (ReHo), has been consistently reported in patients with schizophrenia (SCZ) and their unaffected siblings. To date, little is known about the genetic influences affecting the spontaneous neuronal activity in SCZ. *DISC1*, a strong susceptible gene for SCZ, has been implicated in neuronal excitability and synaptic function possibly associated with regional spontaneous neuronal activity. This study aimed to examine the effects of *DISC1* variations on the regional spontaneous neuronal activity in SCZ.

**Methods:** Resting-state fMRI data were obtained from 28 SCZ patients and 21 healthy controls (HC) for ReHo analysis. Six single nucleotide polymorphisms (SNPs) of *DISC1* gene were genotyped using the PCR and direct sequencing.

**Results:** Significant diagnosis × genotype interactions were noted for three SNPs (rs821616, rs821617, and rs2738880). For rs821617, the interactions were localized to the precuneus, basal ganglia and pre-/post-central regions. Significant interactive effects were identified at the temporal and post-central gyri for rs821616 (Ser704Cys) and the inferior temporal gyrus for rs2738880. Furthermore, *post-hoc* analysis revealed that the *DISC1* variations on these SNPs exerted different influences on ReHo between SCZ patients and HC.

**Conclusion:** To our knowledge this is the first study to unpick the influence of *DISC1* variations on spontaneous neuronal activity in SCZ; Given the emerging evidence that ReHo is a stable inheritable phenotype for schizophrenia, our findings suggest the DISC1 variations are possibly an inheritable source for the altered ReHo in this disorder.

## Introduction

Schizophrenia (SCZ) is a severe and devastating neurodevelopmental disorder with a wide range of clinical clusters and fits a complex mode of inheritance with thousands of genetic variations with small effects (1, 2). It is proposed that distinct schizophrenia-related single nucleotide polymorphisms (SNPs) may be associated with subsets of heritable phenotypes or endo-phenotypes. Recent studies have applied genetic-imaging approach to assess the association of genetic variations with brain morphology and function as such endo-phenotypes in SCZ (3–6). The Regional Homogeneity (ReHo) (7), measuring the local synchronization of neuronal activity at rest, has been successfully applied to fMRI studies in SCZ, majority of which documented decreased ReHo in wide-spread areas including prefrontal, temporal, cingulate, precuneus, and occipital gyri (8–15). Notably, researches using the ReHo have consistently showed that the incoherent neuronal activity was shared by SCZ patients and their healthy siblings (11, 16, 17), implying the incoherence of spontaneous neuronal activity in SCZ is highly associated with inheritable factors. However, owing to the current literatures that few studies, up to date, have examined these inheritable factors for ReHo, the neural mechanism by which the genetic mutants contribute to the altered spontaneous neuronal activity in SCZ remains unknown.

Disrupted-in-Schizophrenia-1 (*DISC1*), a strong susceptible gene for SCZ (18–20), has been shown to be involved in multiple neural processes such as the neurite extension, neuronal proliferation, migration (21–24), and synaptic plasticity within various brain areas (25–27), independently or interactively with other genes such as *NUDEL, YWHAE* (rs28365859), and et al (28–31). Particularly, recent studies have established robust evidence for the involvement of the *DISC1* in neuronal excitability and synaptic functioning (32, 33). For example, knockdown of *DISC1* in rats has been shown to regulate surface levels of the AMPA-type glutamate receptor subunit GLUR1, and the frequency of miniature excitatory postsynaptic currents in cortical neuron (34); Another study reveals that knockdown of *DISC1* in mice leads to accelerated formation of dendritic spines in newborn neurons that have both glutamatergic and GABAergic synapses in the dentate gyrus (35); Moreover, the influence of *DISC1* on synapse function has also been evidenced by one postmortem study using light and electron microscopic approach, which demonstrates that *DISC1* localizes at postsynaptic structures highly associated with synapse functioning in both symmetric and asymmetric synapses (36). Taken together, these studies are suggestive of a regulatory role of the *DISC1* in synaptic functioning (especially for the glutamatergic neuron) which is highly associated with the spontaneous neuronal activity in local brain areas. Combining the evidence of the abnormal spontaneous neuronal activity (measured with ReHo) in SCZ patients and their siblings, it is possible that *DISC1* may be involved in the genetic mechanism of SCZ through its effect on the spontaneous neuronal activity.

Previous studies have indicated the involvement of *DISC1* variations in the brain morphological alteration (4, 37–39) and dysfunction during cognitive tasks associated with SCZ (6, 40, 41). Notably, a prior work by our group found that six *DISC1* SNPs were significantly and consistently associated with the morphological and functional abnormalities of precuneus, and that the precuneus gray matter loss was related to the symptom severity in SCZ patients (42). What should be noted is that our prior work recruited a patient sample with a relatively chronic illness duration (18.1 ± 15.9 years). Given the evidence that long hospitalization, medication and environmental stimulus such as stigma and living place may influence the gene expression through epigenetic processes (43, 44), the present study only recruited a patient sample in the early-stage of SCZ (illness duration < 5 years) (45, 46), although which somewhat overlaps with the prior work. The present study, according to our knowledge, is the first study aiming to investigate the genetic influences of *DISC1* polymorphisms on the resting-state spontaneous neuronal activity (measured with ReHo) in SCZ.

## Materials and methods

### Participants

A total of 28 patients at the early-stage (45, 46) of SCZ were recruited through the Institute of Mental Health, Second Xiangya hospital of Central South University, Changsha, China. Twenty-one healthy controls (HC) were recruited from Changsha city area. All participants were right-handed and no other contraindications to fMRI scanning (e.g., no cardiovascular and metallic implants). All patients were diagnosed with SCZ according to the Structural Clinical Interview for DSM-IV, Patient version (SCID-I/P). The Positive and Negative Syndrome Scale (PANSS) (47) was used as instruments of clinical assessment. Exclusion criteria for participants were neurological or comorbid psychiatric disorders (Axis I or Axis II), history of head injury, other serious illness, alcohol or substance dependence, exposure to electroconvulsive therapy, pregnant or breastfeeding (HC with a history of SCZ or a family history of psychosis were also excluded). All HC were well matched with the SCZ in terms of gender (χ^2^ = 0.458, *P* = 0.498) and years of education (*t* = 0.000, *P* = 1.000), except for age (*t* = −2.939, *P* = 0.005). Differences in demographic details (age, gender, education) were also examined across the genotype groups. Informed consent was given by all participants and the study was approved by the Ethics Committee of the Second Xiangya Hospital, Central South University.

### Genotyping

DNA was extracted from whole venous blood samples. Since our prior work by Gong et al has identified 6 DISC1 SNPs (rs3738401, rs2738880, rs1535530, rs821616, rs821617, and rs12133766) that are consistently associated with resting-state functional alterations in schizophrenia patients, this study focused on the contribution of these SNPs to the abnormality of regional homogeneity at rest in this severe mental disorder. We genotyped *DISC1* SNPs using the PCR and direct sequencing (42). After sequencing, these six SNPs were identified with minor allele frequency > 5% in our sample. Based on our prior work (42), the genotypic groups were divided based on the dominant model: T-allele carriers vs. A homozygotes for rs821616; G-allele carries vs. A homozygotes for rs821617; G-allele carriers vs. A homozygotes for rs2738880; A-allele carriers vs. G homozygotes for rs3738401; C-allele carriers vs. T homozygotes for rs1535530; A-allele carriers vs. G homozygotes for rs12133766. The number of subjects of each genotype for each given SNP was listed in the Table S1.

### MRI data acquisition and image preprocessing

All subjects underwent functional MRI scanning using 1.5-T GE Signa Twinspeed MR scanner (General Electric Medical System, Milwaukee, USA). The participants were informed to lay supine in the scanner with their heads fixed with foam pads and a belt and remain motionless with eyes closed. Gradient-echo echo planar imaging (EPI) was used to acquire resting-state functional images with the following parameters: repetition time/echo time (TR/TE) = 2,000/40 ms, 33 axial slices, 24 × 24 matrix, 90° flip angle, 5 mm section thickness, 1 mm slice gap. For each subject, fMRI scanning lasted for 6 min and 180 volumes were obtained. The fMRI data preprocessing was conducted by SPM8 (University College London, UK; http://www.fil.ion.ucl.ac.uk/spm) and DPARSF (http://restfmri.net/forum/DPARSF). The first 10 volumes of each functional time series were discarded for signal equilibrium and participants' adaptation to the scanning noise. The remaining 170 volumes were analyzed. The steps included slice timing, head-motion correction, spatial normalization in Montreal Neurological Institute (MNI) space and resampling with 3 × 3 × 3 mm^3^ resolution. The head motion of all subjects was <2.0 mm maximum displacement in any direction of x, y, and z and 2.0° in any angular dimension, then band-pass filter (0.01–0.08 Hz) were conducted to reduce low-frequency drift and high-frequency noise; finally, nuisance covariates, including the global mean signal, white matter, and cerebrospinal fluid signals were regressed out.

### ReHo analysis

ReHo maps were calculated using the Kendall's coefficient of concordance (KCC) of the time series within a 27-voxel cubic neighborhood (7), KCC was computed using a cubic cluster size of 27 voxels based on the assumption that a voxel was temporally similar to those of its neighbors, a high ReHo value implies that the resting-state time series have high synchronization with those of its nearest neighbors (26 voxels). To reduce the influence of individual variations in the KCC value, normalizations of ReHo maps were done by dividing the KCC among each voxel by the averaged KCC of the whole brain. Finally smoothed with a Gaussian kernel of 4 × 4 × 4 mm full-width at half maximum (FWHM), we obtained a ReHo map of each subject for statistical analysis.

### Statistical analysis

Demographic and clinical data were analyzed using SPSS, version 19.0 (SPSS, Inc., Chicago, IL), fMRI data analysis was performed using Statistic Parameter Mapping 8 software (SPM8; www.fil.ion.ucl.ac.uk/spm). Deviation of the genotype counts from the Hardy–Weinberg equilibrium (HWE) was tested using a chi-square goodness-of-fit test. Statistical differences in genotypic between SCZ and HC were evaluated by the Chi-square test at significance level of *P* < 0.05. The linkage disequilibrium (LD) analysis was applied to detect the internal relationship of SNPs. In this study, demographic data (age, gender, and education) across diagnosis and genotypes were compared by either two sample *T*-test or χ^2^ test. A 2 × 2 full-factorial model was performed using SPM8 (http://www.fil.ion.ucl.ac.uk/spm), with diagnosis and genotype as between-subject factors by including age and gender as covariates. This full-factorial model allowed us to characterize the main effect of diagnosis, genotype, and diagnosis × genotype interactive effect on the spontaneous neuronal activity. AlphaSim correction (as provided in the REST toolbox) (48) based on the Monte Carlo simulation, was conducted with a combined threshold of *P* < 0.005 at voxel level and *P* < 0.001 at cluster level (at least 30 voxels), which were applied to statistical maps derived from the full factorial model (48–50). We have listed the number of voxels and the smoothness sizes corresponding to the main effect and interactive effect of each given SNP after AlphaSim correction in the Table S2. The ReHo values were automatically calculated and extracted from the regions of interests, and then *post-hoc* analysis was performed to investigate the simple effects of these factors (disease status and genotypes). For further verifying our findings, a permutation-based nonparametric test using the Randomize tool in FSL (http://fsl.fmrib.ox.ac.uk/fsl/fslwiki/Randomize) was performed with family-wise error (FWE) correction (*P* < 0.05) for multiple comparisons corrections. A two-way ANCOVA within general linear model (GLM) framework was performed to calculate the main effect of disease and genotypes as well as the interactive effects between the two factors. All GLM designs included age and sex as covariates. For each GLM, *P*-values were calculated employing permutation-based statistics (10,000 permutations) (51, 52) for multiple comparisons correction. Pearson correlations were calculated between ReHo values and behavioral data including PANSS scores, illness duration and medication dosage in SCZ. Significance was set at *P* < 0.05.

## Results

### Demographic and genotypic characteristics in our sample

Demographic and clinical data were summarized in Table 1. No significant differences were found between SCH and HC group in terms of gender, education, except for the age (*P* < 0.05) which was entered as the covariate into further fMRI data analysis. The genotypes allele distribution did not deviate from Hardy–Weinberg equilibrium (HWE) within the group (SCZ or HC) and with the groups combined (*P* > 0.05) for four SNPs (except for the SNPs rs3738401 and rs12133766 due to their being unsatisfactory with HWE). The genotype groups did not significantly differ with respect to age, gender, and education for the survived four SNPs (*P* > 0.05). Linkage disequilibrium (LD) analysis of SNPs was tested using Haploview software. *R*^2^ for each pair of SNPs were calculated. No other pairwise SNPs showed a high level of LD except the pair of rs821616 and rs821617 (*R*^2^ = 0.8, *P* < 0.001).

**Table 1:** Demographic and clinical characteristics of SCZ and HC.

**Variables**	**SCZ**	**HC**	***P*-value**
Number	28	21	
Age(years)	23.9 (5.4)	28.8 (6.1)	0.005^*^
Gender (M/F)	16/12	14/7	0.498
Education (years)	12.9(2.0)	12.9 (3.8)	1.000
Illness Duration (months)	15.1 (14.2)	–	–
MD (clz eq^a^) (mg/d)	384.9 (243.8)	–	–
**SNPs**			
Rs821617 (G+/AA)	6/22	7/14	0.350
Rs821616 (Cys+/SerSer)	9/19	6/15	0.788
Rs2738880 (G+/AA)	18/10	10/11	0.243
Rs1535530 (C+/TT)	14/14	11/10	0.869
PANSS total score	85.7 (19.9)	–	–
PANSS positive score	17.8 (6.5)	–	
PANSS negative score	21.0 (6.2)	–	
PANSS general score	38.9 (9.4)	–	

Data are given as mean (standard deviation)

* *P < 0.05*.

a *clz eq, chlorpromazine equivalents; MD, medication dose; SCH, schizophrenia; HC, healthy controls; PANSS, positive and negative syndrome scale*.

### Interactions and simple effects between diagnosis and genotype groups

**Figure 1:**
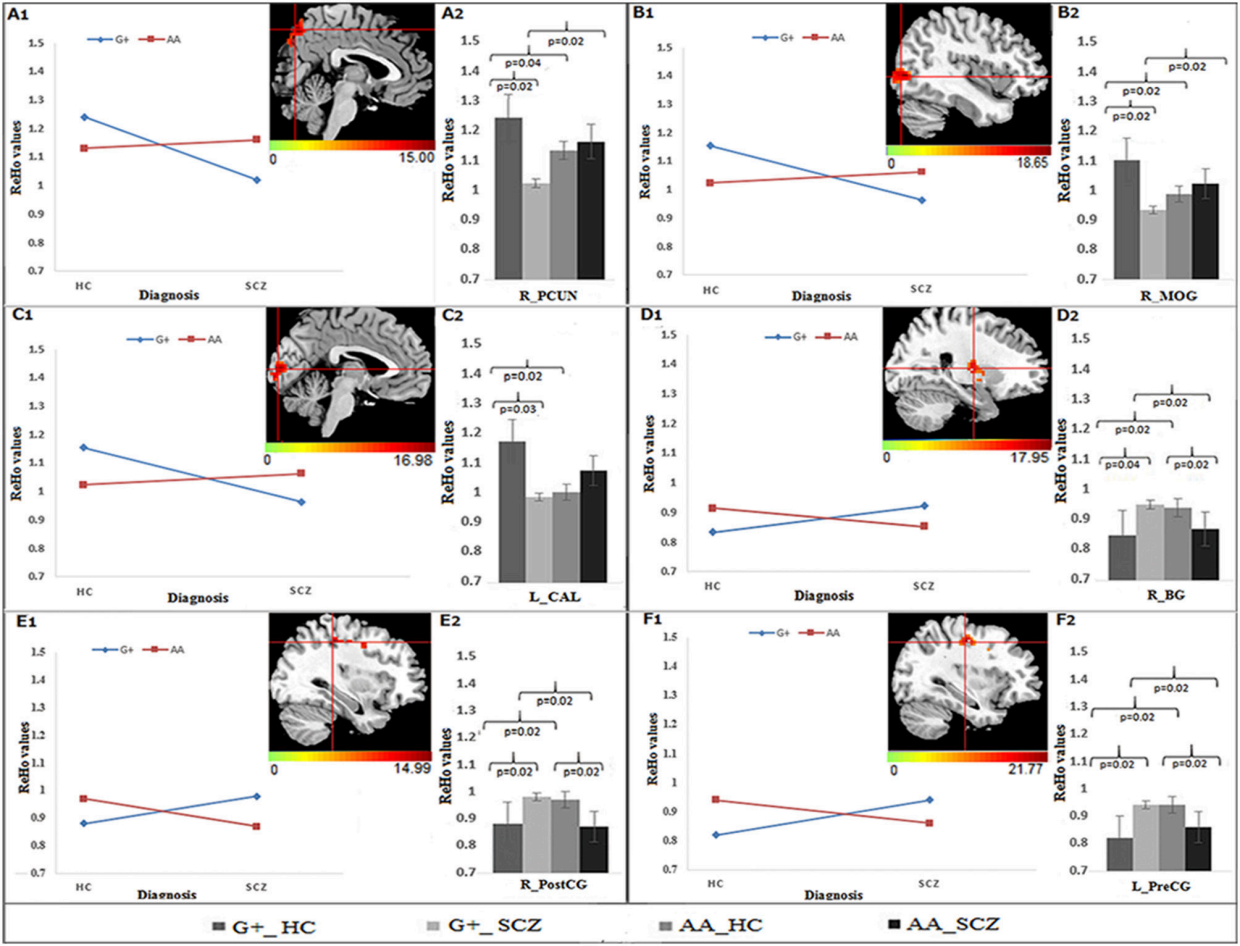
The interaction and simple effect between rs821617 genotypes and diagnosis on ReHo. **(A1–F1)** Clusters of significance for the interaction. **(A1)** R-PCUN, **(B1)** R-MOG, **(C1)** L-CAL, **(D1)** R-BG, **(E1)** R-PostCG, **(F1)** L-PreCG. **(A2–F2)** Plots show the simple effect resulted from the genotype G+ vs. AA across diagnosis.

As shown in the Table 2, significant group × genotype interactive effects on the ReHo were found for three SNPs (rs821617, rs821616, rs2738880). For rs821617, a significant interaction was found in the right precuneus (PCUN), middle occipital gyrus (MOG), basal ganglia (BG), post-central gyrus (PostCG), and left pre-central gyrus (PreCG), calcarine (CAL) (Figures 1A1–F1). Further *post-hoc* analysis indicated that for G allele carriers, SCZ showed lower ReHo in the right PCUN (Figure 1A2), MOG (Figure 1B2), and left CAL (Figure 1C2) comparing to HC, while no significance of ReHo was observed between A homozygous SCZ and HC group; Meanwhile, the G-allele carriers showed lower ReHo in the right PCUN, MOG, and higher ReHo in the right BG (Figure 1D2), PostCG (Figure 1E2), and left PreCG (Figure 1F2) than the AA carriers in SCZ group, whereas the HC group showed the opposite findings in the above regions.

**Table 2:** Main effect and interactive effects across diagnosis and genotypes.

**Contrast**	**Cluster size**	***P*-value**	**Effect size (partial η^2^)**	**MNI coordinates**			**Region**
				**x**	**y**	**z**	
**MAIN EFFECT OF DIAGNOSIS**							
	112	1.00*E*−06	0.40	−15	−15	12	THA (VLN)
	55	1.40*E*−05	0.34	12	−12	9	THA (VLN)
**MAIN EFFECT OF GENOTYPES**							
rs821617	31	1.80*E*−05	0.33	42	15	54	MFG
rs821616	69	2.00*E*−06	0.39	−30	36	48	MFG
**INTERACTION: GENOTYPE** × **DIAGNOSIS**							
rs821617	101	3.00*E*−05	0.33	3	−75	51	PCUN
	53	4.40*E*−05	0.32	42	−81	0	MOG
	115	2.60*E*−04	0.26	−3	−93	6	CAL
	41	2.90*E*−05	0.33	24	−9	9	PUTA
	38	2.90*E*−05	0.33	21	−3	−6	PALL
	91	8.10*E*−05	0.30	−33	−18	45	PreCG
	116	2.45*E*−04	0.27	33	−33	45	PostCG
rs821616	35	2.60*E*−05	0.33	−60	−51	9	MTG (extending to STG)
	40	2.30*E*−05	0.34	−39	−42	63	PostCG
rs2738880	63	1.80*E*−05	0.35	−51	−54	−6	ITG

*AlphaSim correction with a combined statistical thresholds of P < 0.005 at voxel level and P < 0.001 at cluster level. THA, thalamus; MFG, middle frontal gyrus; PCUN, precuneus; MOG, middle occipital gyrus; CAL, calcarine; PUTA, putamen; PALL, pallidum; ITG, inferior temporal gyrus; STG, MTG, superior/middle temporal gyrus; PreCG, Pre-central gyrus; PostCG, Post-central gyrus; VLN, Ventral Lateral Nucleus*.

**Figure 2:**
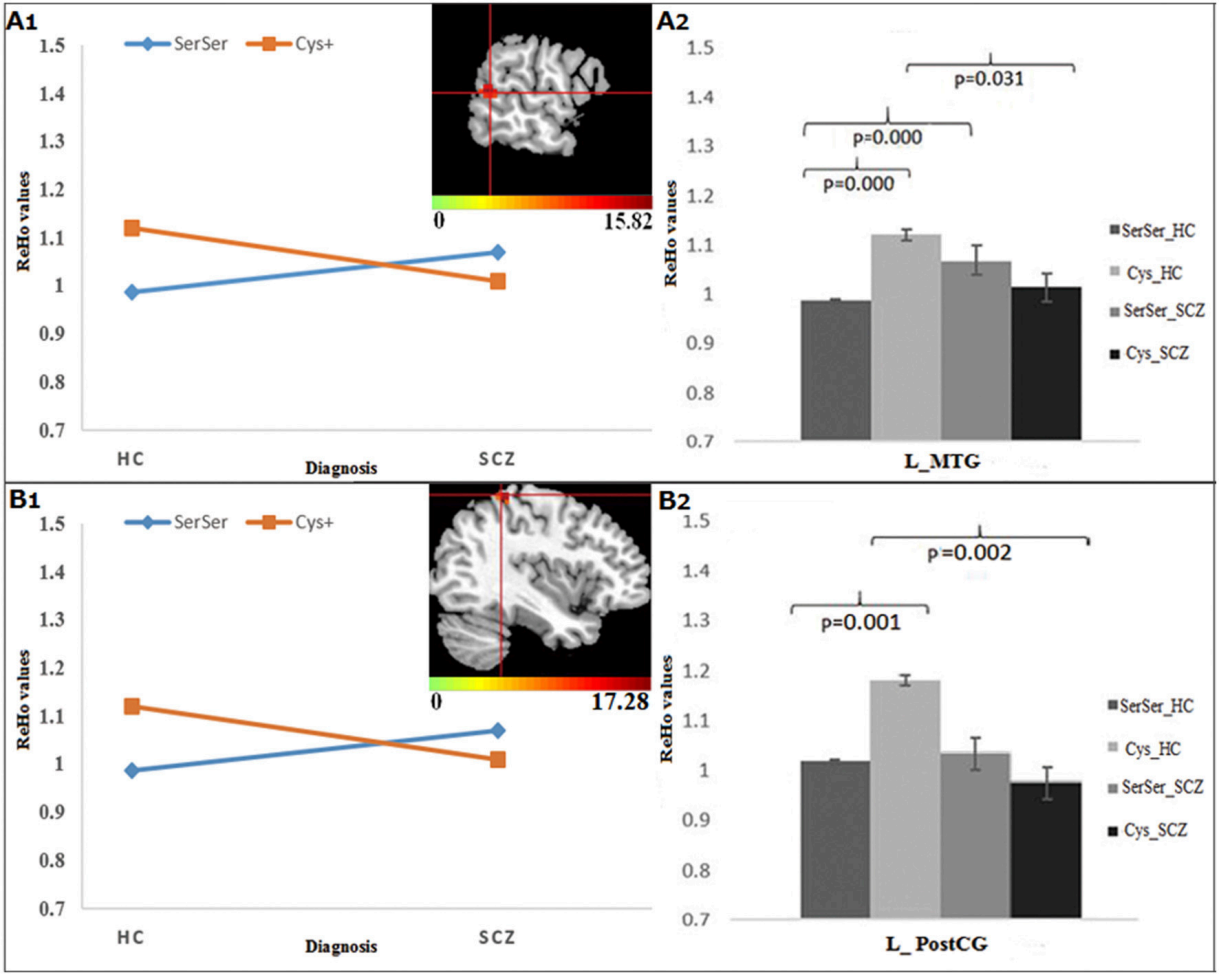
The interaction and simple effect between Ser704Cys genotypes and diagnosis. **(A1,B1)** Clusters of significance in the L-MTG, STG, and PostCG. **(A2,B2)** The simple effect of Ser704Cys in these clusters.

For Ser704Cys, the genotype × diagnosis interactions were found in the left middle temporal gyrus (MTG), extending to superior temporal gyrus (STG) (Figure 2A1) and PostCG (Figure 2B1). Further *post-hoc* analysis showed that for Cys allele carriers (Figures 2A2,B2), SCZ group showed lower ReHo in the left MTG and PostCG compared to HC group, while for Ser homozygous SCZ group showed higher ReHo in the left MTG than HC group; Meanwhile, in the HC group, the Ser homozygotes showed lower ReHo than the Cys-allele carriers in the left MTG and PostCG, which was not observed in the SCZ group.

**Figure 3:**
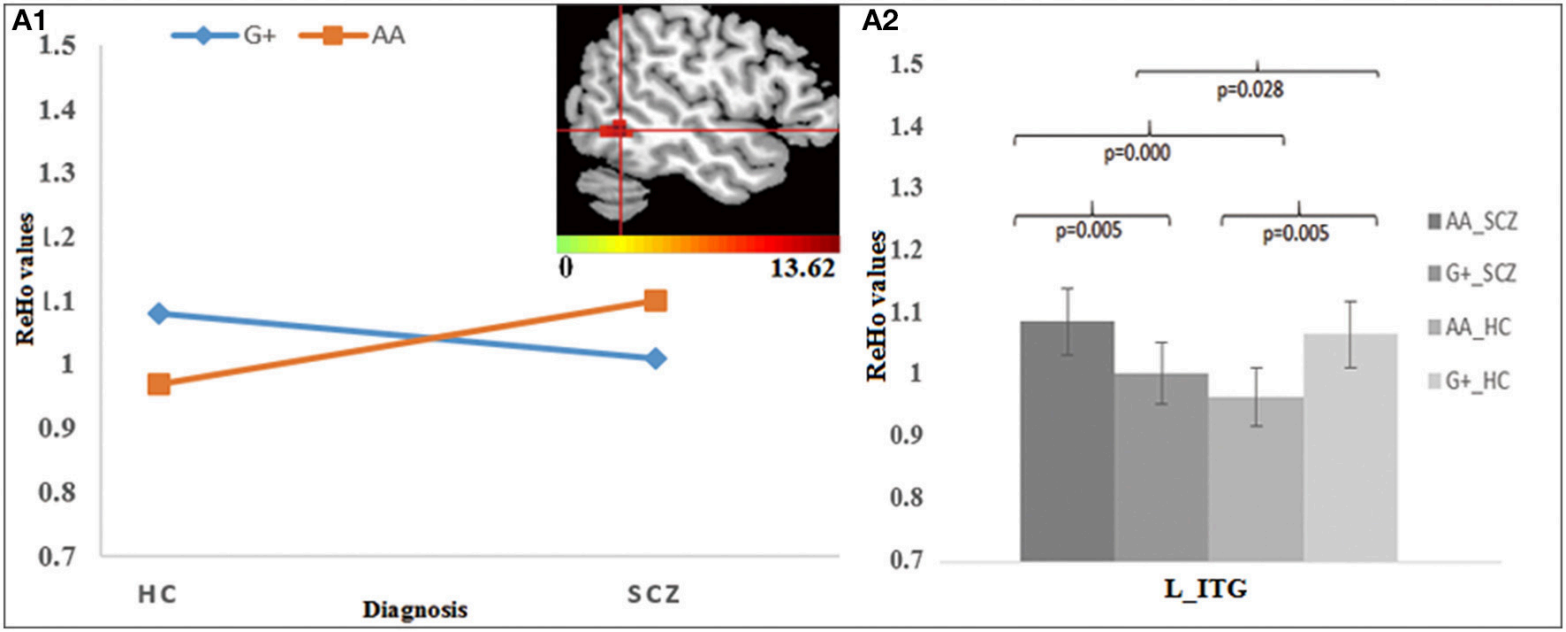
The interaction and simple effect between rs2738880 genotypes and diagnosis. **(A1)** Cluster of significance for the interaction effect in the L-ITG. **(A2)** The simple effect in the L-ITG.

For rs2738880, the genotype × diagnosis interaction was found in the left inferior temporal gyrus (ITG) (Figure 3A1). Further *post-hoc* analysis (Figure 3A2) showed that for the G-allele carriers, SCZ group showed lower ReHo in the left ITG compared to HC, whereas an opposite finding was observed in the A homozygotes; Meanwhile, G-allele carriers showed lower ReHo compared to A homozygotes in SCZ group, while in HC group an opposite pattern was revealed.

### Main effect of *DISC1* genotypes on ReHo across all subjects

**Figure 4:**
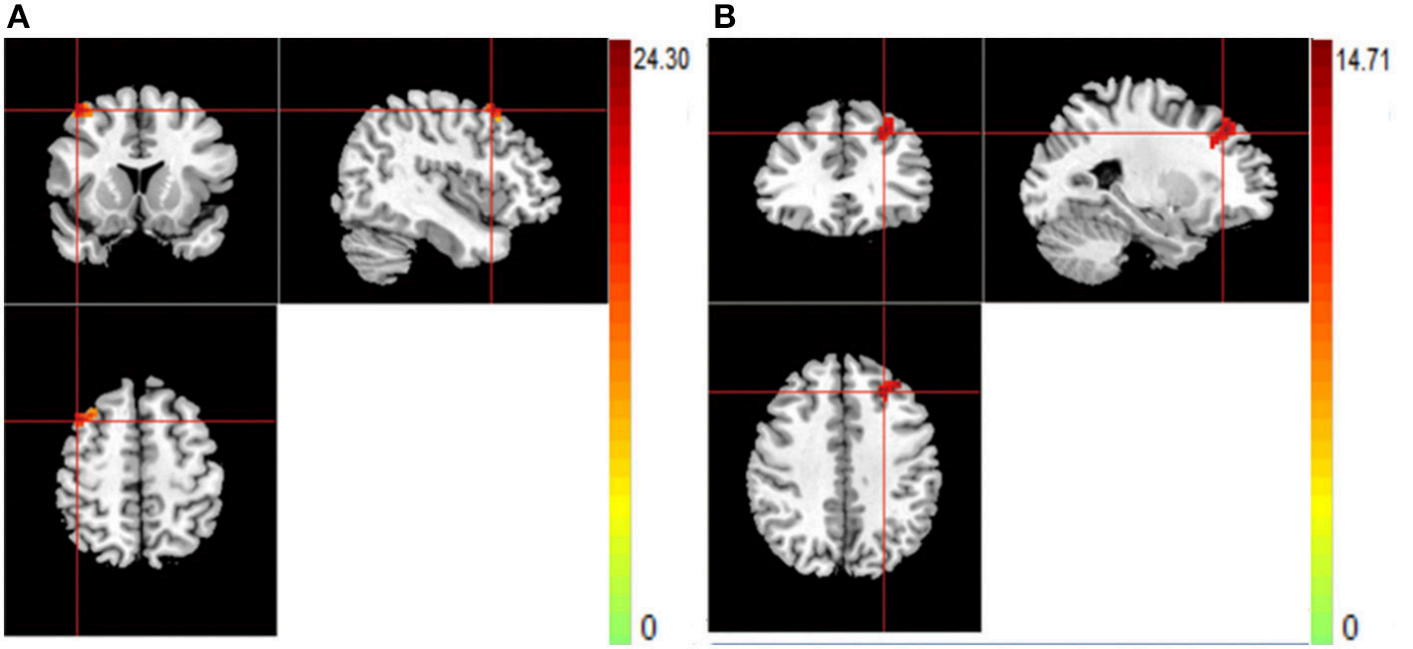
**(A,B)** The images show the main effect of genotypes. The hot color indicate that ReHo is lower when carrying the G-allele of rs821617, T-allele of rs821616 than homozygotes group for all subjects. **(A)** R-MFG for rs821617 **(B)** L-MFG for rs821616.

For rs821617, the G carriers showed lower ReHo in the right middle frontal gyrus (MFG) compared with the A homozygous group (Figure 4A); For Ser704Cys, Ser homozygotes showed higher ReHo in the left MFG than Cys-allele carriers (Figure 4B); For rs2738880 and rs1535530, no significant genotype main effects were found.

### Main effect of diagnosis on ReHo

**Figure 5:**
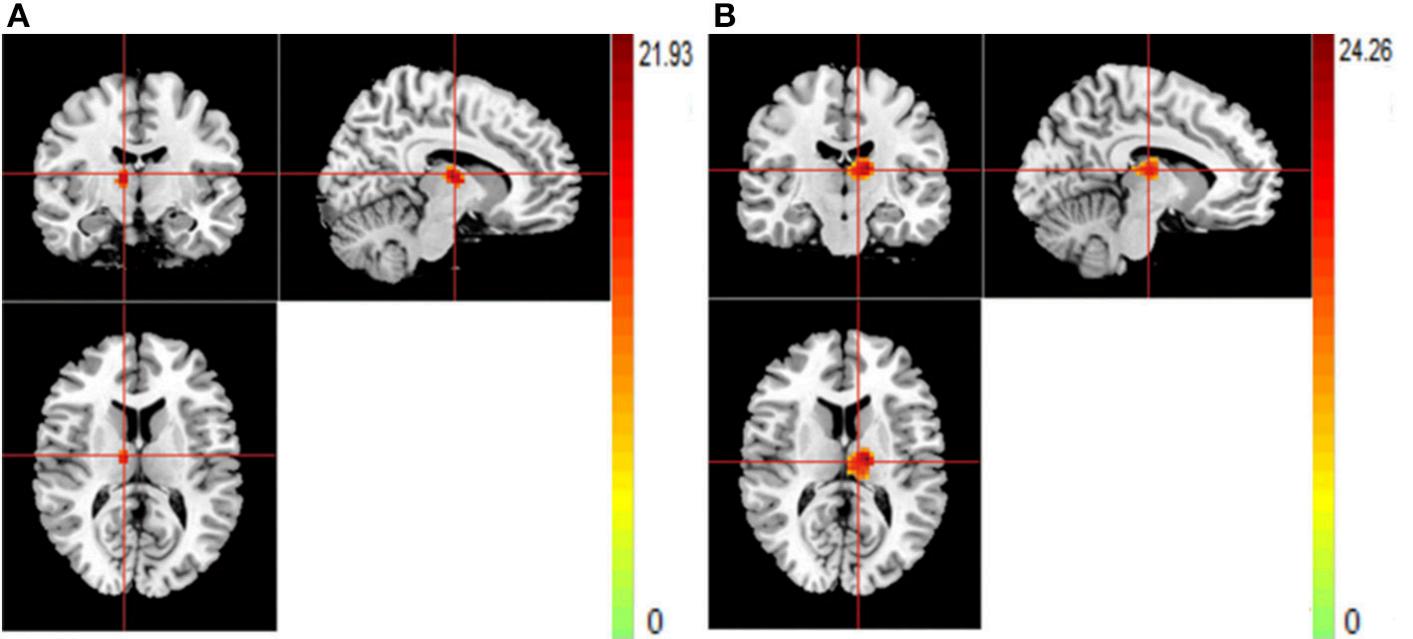
**(A,B)** The images show the main effect of diagnosis. The hot color indicate that ReHo is higher in HC than SCZ for all SNPs. **(A)** R-THA **(B)** L-THA.

Patients with SCZ showed lower ReHo compared to healthy controls in the bilateral thalamus (THA) (Figures 5A,B).

The findings using non-parametric test (*P* < 0.001, uncorrected) were quite similar with our original results (see Table S3). Rs821616 and rs821617 were found to have significant interactive effects of genotypes with diagnosis on the ReHo in distributed brain regions, which located at the temporal gyrus for the rs821616 and the PCUN, MOG, PUTA, PostCG, and PreCG for the rs821617. In addition, a main effect of *DISC1* genotypes was observed in the MFG in all subjects. However, only the interactive effects between rs821617 and diagnosis on the PCUN and visual cortex were survived after FWE correction.

### Correlation

There were no significant correlations of ReHo with severity of symptoms, illness duration, or medicine dosage in SCZ (*P* > 0.05).

## Discussion

This study, according to our knowledge, is the first to document the genetic effect of *DISC1* variations on the resting-state regional neuronal activity in SCZ. Of six *DISC1* SNPs, three (Ser704Cys, rs821617, rs2738880) were found to have significant interactive effects of genotypes with diagnosis on the ReHo in distributed brain regions, which located at the temporal and PostCG for the rs821616, the ITG for the rs2738880, and the PCUN, BG, MOG, CAL, PostCG, and PreCG for the rs821617. Previous studies have reported that the altered spontaneous neuronal activity (measured with ReHo) in those regions above was shared between SCZ patients and their unaffected siblings (11, 16). Our findings provide the novel evidence that variations of *DISC1* gene may potentially underlie these shared abnormalities associated with SCZ. Further simple genotypic effects revealed in the current study suggest a complicated pattern of genetic influence of *DISC1* variations on the resting-state neuronal activity. In addition, a main effect of *DISC1* genotypes was observed in the MFG in all subjects. No significant correlations of ReHo with severity of symptoms in SCZ.

The *DISC1* Ser704Cys SNP has been widely studied in human beings, identifying a close relationship of this allele with brain morphology and functioning which are impaired in SCZ (37, 38, 40, 41, 53, 54). Notably, one volumetric study has identified significant diagnosis (SCZ vs. HC) × genotype (Cys+ vs. SerSer) interaction on the gray matter volumes in the frontal and temporal cortices (54); another task-induced fMRI study also showed diagnosis × genotype interaction on brain activation in the frontal cortex during a verbal fluency task (6). The evidence together indicates that genetic variation of the *DISC1* Ser704Cys may relate to the risk of SCZ and interplay with the disease for abnormal brain morphology and task-oriented functions. The present study found the interactive effects of Ser704Cys with diagnosis on the ReHo in the MTG (extending to the STG) and PostCG, further demonstrating the involvement of this SNP in the abnormal resting-state neuronal activity associated with SCZ.

In particular, the MTG and STG, responsible for comprehension and conceptual or semantic processing (55), have been consistently documented to be critical in the neuropathology of psychotic symptoms (especially for hallucinations) in SCZ (56). One fMRI study showed that the altered activation in the temporal gyrus in the siblings of SCZ during the N-back task (57). Most importantly, abnormal ReHo in these regions has been observed to be shared by SCZ patients and their unaffected siblings (11), suggesting the inheritable influence on the functional activity of the MTG and STG in SCZ. Our findings provide the first evidence that *DISC1* Ser704Cys mutations may be the genetic source for this inheritable influence.

Rs821617, has a tight linkage with rs821616 leading to the change of amino acid (K800R) in *DISC1* protein isoform b (NM001164538; http://www.ncbi.nlm.nih.gov/protein/NP_001158010.1). A prior study by our group in chronic patients reported the involvement of this locus in the development of SCZ, documenting that a significant association between rs821617 and abnormal functional connectivity of the PCUN with frontal cortex in SCZ (42). Consistently, this study found a significant interaction of this SNP with diagnosis on the resting-state PCUN activity. This consistent finding across chronic and early-stage patients may suggest that the influence of DISC1 variation (especially for the rs821617) may be not modulated by the long hospitalization, medication and other environmental factors such as stigma, living place (43, 44). Future neuroimaging-genetic studies using longitudinal design will be verify this notion.

The PCUN, broadly known as the key node in the so-called “default mode network” (DMN) (58), is associated with episodic memory, self-referential processing, and visuo-spatial imagery (59–61), which are all consistently observed to be impaired in SCZ, such as the self-processing (62) and insight (63). Functional alteration in this region has been repeatedly found in unaffected siblings of SCZ across cognition-related state (57, 64, 65) and resting-state (66). Notably, by applying the ReHo, a recent fMRI study reported altered resting-state neuronal activity in the PCUN in healthy siblings of SCZ (16), which supports that the PCUN dysfunction may be a potential neuroimaging endophenotype for SCZ. Our finding may further reveal that genetic underpinning of this endophenotype is associated with the *DISC1* rs821617 polymorphisms. Most interestingly, our further simple effects showed that in G-carriers, but not in A homozygotes, SCZ patients exhibited reduced ReHo relative to HC in the PCUN, suggesting the G allele of this SNP may be engaged in the neuropathology of SCZ through its specific influence on the resting-state PCUN activity.

Another key region identified as related with the rs821617 is the BG (putamen and pallidum), which is well-known for its dopaminergic hyperfunction associated with the biological mechanism of SCZ. Inheritable contributions to the BG functional and structural abnormalities have been also revealed in previous studies involving SCZ patients and their unaffected siblings (67–71), which may be originated from the *DISC1* rs821617 mutations observed in this study. The role of the *DISC1* gene in regulating dopaminergic function (72) may partly explain the *DISC1* rs821617 mutations affect the BG resting-state neuronal activity in SCZ.

Additionally, we also found a significant interaction between rs2738880 genotype and diagnosis on the ReHo in the ITG, which is a key region responsible for language processing, working memory, social cognition and emotional visual processes. The morphological and functional alterations have been consistently found in SCZ patients (73–76) and their healthy siblings (76), as well as the subjects with psychosis risk syndrome (PRS) (12). As a rare variation in intron region upstream the exon 9 of DSC1 gene, the rs2738880 was examined in only one study (42) and its exact mechanism on brain activity still remains unclear.

Despite the interactions between diagnosis and *DISC1* genotypes on the resting-state neuronal activity, our findings also showed informative simple effects. These complicated simple effects may be characterized by two features. Firstly, the *DISC1* mutations may exert different genetic effects on ReHo between SCZ and HC. For rs821617 and rs2738880, the genotype effects on ReHo in SCZ are completely opposite to that in the HC, while the significant rs821616 genotype effects are observed in HC, but not in SCZ. These findings echo with previous studies (6, 77) and comply with the notion that SCZ fits a complex mode of inheritance with numerous genetic variations (19, 78), where the effects of numbers of alleles combine to form a continuum of internal phenotypic variation in brain function in SCZ. However, how *DISC1* interacts with other genes or environmental factors to influence the resting-state neuronal activity in SCZ still calls for future studies. Secondly, we observed that in rs821616 T-allele carriers, but not in A homozygotes, SCZ exhibited reduced ReHo in the PostCG relative to HC, interestingly, the same pattern was also observed in rs821617 G-allele carriers. These findings imply that the T allele of rs821616 and the G allele of rs821617 may be engaged in the neuropathology of SCZ through their specific influences on the resting-state neuronal activity.

Robust biological evidence has supported the critical role of *DISC1* gene in the brain morphology and functioning. Traditionally, *DISC1* protein is suggested to be essential in neurite outgrowth and neuronal migration (21–24, 36), recent evidence implicates that mutations of *DISC1* disrupt synapse formation, regression and function, finally leading to the dysfunctional neurotransmission and altered neuronal activity (32, 33), such as the glutamatergic and the dopaminergic pathways, eventually inducing schizophrenia-like symptoms including positive, negative, and cognitive symptoms (34, 72). The present study, using a genetic-neuroimaging approach *in vivo*, extends previous evidence to show a significant influence of *DISC1* mutations on the regional spontaneous neuronal activity in SCZ patients.

Several limitations should be noted in this study. First of all, our sample is relatively small. For the purpose of further verifying our results, we did a non-parametric test using 10,000 permutations on our neuroimaging-genetic data and showed similar findings with our prior results. Most importantly, the interactive effects between rs821617 and diagnosis on the precuneus and visual cortex still survived after FWE correction, increasing the reliability of our findings. However, these findings in the present study need to be verified and replicated in future studies with a relatively large sample; Secondly, no correlations between ReHo and symptom severity were observed in this study. The study by Gong et al. also did not find associations between functional alterations and symptoms, but found a correlation of gray matter loss of precuneus with negative symptoms. The null finding of correlation analysis may be accounted for by our relatively small sample. However, since previous studies never found the correlations of clinical symptoms with ReHo, another possible explanation is that the synchrony of spontaneous neuronal activity could be used qualitatively to help locate functional alterations, but not as a quantitative marker for evaluating SCZ symptoms. Thirdly, this study recruited the patients in the early stage of SCZ to control the effects of medication and hospitalization on the resting-state neuronal activity, which along with the methodological distinctions (ReHo vs. functional connectivity) may account for the differences between our findings and the prior work by Gong et al. However, the effect of antipsychotic medications and illness duration could not be completely ruled out. Future studies on first-episode drug-naïve patients may put further insight on the relationship of *DISC1* mutants with SCZ brain functional abnormalities. Fourthly, environmental factors including stress and childhood trauma have been proven to interact with genetic variants to contribute to the development of SCZ (79, 80). Interestingly, a recent study found that the mutant *DISC1* mice exposed to a diet containing neurotoxicant (Pb2+) produced the brain and behavior abnormalities consistent with SCZ (81), suggesting *DISC1* variations related to SCZ may be relevant to the environmental xenobiotics. However, this study did not obtain such environmental information in our samples. It still calls for future study to examine how *DISC1* variants interact with risky environmental factors to influence the neurodevelopment of SCZ.

In summary, this study highlights the importance of the *DISC1* polymorphisms in the modulation of resting-state neuronal activity in SCZ. Our findings support that the *DISC1* variations are highly associated with the abnormal resting-state neuronal activity repeatedly observed in SCZ, and potentially, extend the evidence to show the genetic underpinning of the shared alterations of resting-state spontaneous neuronal activity (endo-phenotype) between SCZ and their unaffected siblings. The complicated simple effects suggest that the *DISC1* gene possibly interact with other genes or environmental factors to contribute to the altered resting-state neuronal activity in SCZ, and thus, future studies in the framework of gene-gene or gene-environment interaction are called for to provide further insight into the genetic mechanism of resting-state brain dysfunction in SCZ. Moreover, recent evidence demonstrates that reduced ReHo benefits from 13 weeks paliperidone treatment mainly targeting at the dopaminergic pathways in SCZ patients (82), suggesting that alterations of ReHo may be a potential pharmacological target for SCZ treatment. Our findings possibly provide a means to target specific regions with highest degree of localized connectivity abnormalities for therapeutic purposes in this severe mental disorder.

## Author contributions

WP: designed the study; NG and WP: analyzed, interpreted the data, and wrote the first draft of the manuscript; ZX, ZL, ML, and LP: provided fMRI technical support and revised it critically for important intellectual content. Other authors collected the data and provided assistance for statistical analysis. All authors contributed to and have approved the final manuscript.

### Conflict of interest statement

The authors declare that the research was conducted in the absence of any commercial or financial relationships that could be construed as a potential conflict of interest.
