# Supplementary material for: Effects of DISC1 Polymorphisms on Resting-State Spontaneous Neuronal Activity in the Early-Stage of Schizophrenia
Source: Front Psychiatry. 2018 May 23;9:137. doi: 10.3389/fpsyt.2018.00137 (PMC5974222; doi:10.3389/fpsyt.2018.00137)
Supplement: Supplementary file 1 [file Data_Sheet_1.doc]

**Supplementary Materials for**

**Effects of DISC1 polymorphisms on Resting-State Spontaneous Neuronal Activity in** **the early-stage of schizophrenia**

**Ningzhi Gou^1,2^, Zhening Liu^1,2^, Lena Palaniyappan^3^, Mingding Li^4^, Yunzhi Pan^1,2^, Yicheng Long^1,2^, Xudong Chen^1,2^, Haojuan Tao^1,2^, Guowei Wu^1,2^, Xuan Ouyang^1,2^, Zheng Wang^1,2^, Taotao Dou^5^, Zhimin Xue^1,2^*****, Weidan Pu ^2,6^***

* Correspondence author: e-mail: [pulv1128@126.com](mailto:pulv1128@126.com).

**Table S1** Number of Subjects of each Genotype

| SNP | rs2738880 | | | rs1535530 | | | rs821616 | | | rs821617 | | |
| --- | --- | --- | --- | --- | --- | --- | --- | --- | --- | --- | --- | --- |
| Genotype | GG | GA | AA | TT | TC | CC | TT | TA | AA | GG | GA | AA |
| Total*(N)* | 4 | 24 | 21 | 24 | 23 | 2 | 0 | 15 | 34 | 0 | 13 | 36 |
| Diagnosis |  |  |  |  |  |  |  |  |  |  |  |  |
| SCZ*(N)* | 3 | 15 | 10 | 14 | 13 | 1 | 0 | 9 | 19 | 0 | 6 | 22 |
| HC*(N)* | 1 | 9 | 11 | 10 | 10 | 1 | 0 | 6 | 15 | 0 | 7 | 14 |

*SNP: single nucleotide polymorphism; N: number; SCZ: schizophrenia; HC: healthy controls*

**Table S2 Cluster size and FWHM of each statistical map**

| Contrast | Cluster size | FWHM^a^ | |
| --- | --- | --- | --- |
| **Main effect of diagnosis** |  | |  |
| rs821617  rs821616  rs2738880  rs1535539 | 31  34  47  34 | 5.495  5.687  6.244  5.432 | |
| **Main effect of genotype** |  | |  |
| rs821617 | 31 | 5.719 | |
| rs821616 | 33 | 5.663 | |
| **Interaction: genotype × diagnosis** |  | |  |
| rs821617 | 50 | 6.441 | |
| rs821616 | 35 | 5.690 | |

*^a^**P*＜*0.005 at voxel level and P*＜*0.001 at cluster level with AlphaSim correction.*

*FWHM: full-width at half maximum*

**Table S3** Main effect and interactive effects across diagnosis and genotype using non-parametric test

| Contrast | Cluster size | *F* value | | MNI coordinates  x y z | | | Region |
| --- | --- | --- | --- | --- | --- | --- | --- |
| **Main effect of diagnosis** |  | |  | | | | |
|  | 168 | 13.08 | | -9 | -12 | 6 | THA (VLN) |
|  | 101 | 17.79 | | 18 | -15 | 6 | THA (VLN) |
| **Main effect of genotype** |  | |  | | | | |
| rs821617 | 20 | 8.31 | | 33 | 36 | 21 | MFG |
| rs821616 | 100 | 10.05 | | -33 | 15 | 24 | MFG |
| **Interaction: genotype × diagnosis** |  | |  | | | | |
| rs821617 | 123 | 14.23 | | 21 | -81 | 51 | PCUN* |
|  | 38 | 8.72 | | 33 | -93 | 21 | MOG* |
|  | 23 | 17.38 | | 33 | -18 | 12 | PUTA |
|  | 7  51 | 8.13  6.69 | | 27  -39 | -3  6 | 54  36 | PreCG  PreCG |
|  | 25 | 5.04 | | 54 | -24 | 39 | PostCG |
| rs821616 | 14 | 16.05 | | -63 | -39 | 12 | STG |

*Uncorrected P values (*＜*0.001) were calculated employing permutation-based statistics (10,000 permutations);*

** P*＜*0.05 with FWE correction.*

*THA: thalamus; MFG: middle frontal gyrus; PCUN: precuneus; MOG: middle occipital gyrus; PUTA: putamen;*

*STG: superior temporal gyrus; PreCG: Precentral gyrus; PostCG: Postcentral gyrus; VLN: Ventral Lateral Nucleus*
